# Effect of a bound anion on the structure and dynamics of halorhodopsin from *Natronomonas pharaonis*

**DOI:** 10.1063/1.5125621

**Published:** 2019-10-23

**Authors:** Misao Mizuno, Yumi Shimoo, Hideki Kandori, Yasuhisa Mizutani

**Affiliations:** 1 Department of Chemistry, Graduate School of Science, Osaka University, 1-1 Machikaneyama, Toyonaka, Osaka 560-0043, Japan; 2 Department of Life Science and Applied Chemistry, Nagoya Institute of Technology, Showa-ku, Nagoya, Aichi 466-8555, Japan

## Abstract

Active ion transport across membranes is vital to maintaining the electrochemical gradients of ions in cells and is mediated by transmembrane proteins. Halorhodopsin (HR) functions as a light-driven inward pump for chloride ions. The protein contains all-*trans*-retinal bound to a specific lysine residue through a protonated Schiff base. Interaction between the bound chloride ion and the protonated Schiff base is crucial for ion transport because chloride ion movement is driven by the flipping of the protonated Schiff base upon photoisomerization. However, it remains unknown how this interaction evolves in the HR photocycle. Here, we addressed the effect of the bound anion on the structure and dynamics of HR from *Natronomonas pharaonis* in the early stage of the photocycle. Comparison of the chloride-bound, formate-bound, and anion-depleted forms provided insights into the interaction between the bound anion and the chromophore/protein moiety. In the unphotolyzed state, the bound anion affects the π-conjugation of the polyene chain and the hydrogen bond of the protonated Schiff base of the retinal chromophore. Picosecond time scale measurements showed that the band intensities of the W16 and W18 modes of the tryptophan residues decreased instantaneously upon photoexcitation of the formate-bound form. In contrast, these intensity decreases were delayed for the chloride-bound and anion-depleted forms. These observations suggest the stronger interactions of the bound formate ion with the retinal chromophore and the chromophore pocket. On the nanosecond to microsecond timescales, we found that the interaction between the protonated Schiff base and the bound ion is broken upon formation of the K intermediate and is recovered following translocation of the bound anion toward the protonated Schiff base in the L intermediate. Our results demonstrate that the hydrogen-bonding ability of the bound anion plays an essential role in the ion transport of light-driven anion pumps.

## I. INTRODUCTION

Photoexcitation of microbial rhodopsins leads to various functionalities, such as ion transport across membranes and light sensing. Halorhodopsin (HR) functions as a light-driven inward chloride ion pump across the cell membrane to generate an electrochemical potential gradient. HR contains all-*trans*-retinal as a chromophore bound to a lysine residue (Lys256) through a Schiff base linkage and surrounded by seven transmembrane α helices. To date, photoreactions of two HR homologs, from *Halobacterium salinarum* (HsHR) and from *Natronomonas pharaonis* (NpHR), have been intensively studied. Local structural changes due to the photoisomerization of the retinal chromophore from the all-*trans* to the 13-*cis* form initiate sequential changes in the protein structure on the picosecond to millisecond timescales. These structural changes result in the formation of a series of intermediates designated as the K, L, N, and O intermediates, and recovery of the unphotolyzed state.^1–4^

The binding of anions such as chloride to HR has been studied to elucidate the ion transport mechanism. Crystallographic studies showed that the transported chloride ion is bound in the extracellular vicinity of the protonated Schiff base of the retinal chromophore.^5,6^ Bromide and iodide ions can also be taken up and transported by HR. Crystallographic^7^ and spectroscopic analyses^8–12^ have revealed that monoatomic halide ions can be replaced by polyatomic anions at the anion binding site near the chromophore. For example, when a nitrate ion is bound to HR, the protein pumps the nitrate ion with the same efficiency as it pumps a chloride ion.^9^ The deprotonated M intermediate formed in the photocycle in the presence of azide and formate ions has features similar to those of bacteriorhodopsin (BR), resulting in the conversion of HR into a proton pump.^11,12^ The formation of the M intermediate in the HR photocycle results from the lowered p*K*_a_ value of the protonated Schiff base upon replacement of the bound anion with a polyatomic anion such as an azide or formate ion.^12^

We recently reported the structural evolution of the retinal chromophore in the photocycle of NpHR using time-resolved visible resonance Raman (RR) spectroscopy on the nanosecond-millisecond time scales.^13^ Resonance enhancement of the vibrational bands of the retinal chromophore under visible Raman excitation conditions allowed us to determine the structures of the retinal chromophore in the unphotolyzed state and of the photointermediates, shedding light on the effect of the halide ion on the chromophore's structure. Replacement of the chloride ion with a bromide or iodide ion has a limited effect on the chromophore structure in the HR photocycle because these ions are congeners.

In this study, we carried out comprehensive time-resolved RR measurements on HR bound to anions with very different proton affinities on the picosecond to microsecond timescales to reveal structural changes in the chromophore pocket of NpHR. We examined the anion dependence on the structural changes by measuring the visible RR spectra of three forms of NpHR: chloride-bound NpHR functioning as a chloride-ion pump, formate-bound NpHR showing a proton-pump-like photocycle,^12^ and anion-depleted NpHR lacking a pumped anion at the binding site near the retinal chromophore. In addition, we used picosecond time-resolved ultraviolet RR (UVRR) spectroscopy to selectively measure Raman spectra of the tryptophan and tyrosine residues in NpHR. This is a powerful technique for revealing the initial protein response to chromophore isomerization, as demonstrated previously using several microbial rhodopsins.^14–18^ In the previous studies, we reported the protein response for cation (proton)-pumping^14^ and photosensory rhodopsins.^15–17^ Here, we report the protein response for the anion-pumping rhodopsin for the first time using UVRR spectroscopy and show similarities in rates of the structural changes among these rhodopsins. The present study provides insights into the interaction between the bound anion and the chromophore/protein in the early stages of the NpHR photocycle, as well as in the unphotolyzed state. The interaction between the protonated Schiff base and the bound ion formed in the unphotolyzed state is disrupted upon formation of the K intermediate and is recovered upon translocation of the bound anion toward the protonated Schiff base in the L intermediate. The formate ion forms a stronger interaction than the chloride ion, which accelerates formation of the L intermediate. The present results demonstrate that the hydrogen-bonding ability of the bound anion plays an essential role in the ion transport of light-driven anion pumps.

## II. MATERIALS AND METHODS

### A. Sample preparation

The NpHR sample was prepared as described previously,^19^ with some modifications. Briefly, *Escherichia coli* BL21(DE3) harboring a plasmid encoding NpHR with a histidine tag at the C-terminus was grown in 2 × YT medium in the presence of 50 *μ*g/ml ampicillin and 10 *μ*M all-*trans*-retinal following induction with 1 mM isopropyl-β-D-thiogalactopyranoside. The gray-colored cells were sonicated and the cell membranes were solubilized with 1% (w/v) *n*-dodecyl-β-D-maltoside (DDM) (Annatrace, D310S). The solubilized membranes were purified with a Ni-affinity column (GE Healthcare, HisTrap HP) and an ion-exchange chromatography column (GE Healthcare, HiTrap Q HP). Then, the samples were suspended in a buffer solution comprising 10 mM 3-(*N*-morpholino)propanesulfonic acid (MOPS)-NaOH (pH 7.0) and 0.1% (w/v) DDM by ultrafiltration. The buffer solutions contained 150 mM NaCl and 1 M HCOONa for the chloride-bound and the formate-bound NpHR samples, respectively. The buffer solution for the anion-depleted NpHR samples contained no additional salt. The concentration of NpHR was generally approximately 35 μM. The NaCl concentration was chosen based on the dissociation constant, *K*_d_, for NaCl reported previously.^19^ The HCOONa concentration was determined from *K*_d_ obtained by the titration experiment described in Fig. S1 in the supplementary material. Na_2_SO_4_ was added as an internal intensity standard for the Raman measurements because sulfate does not bind the protein.^10^

We measured the deuteration effect by exchanging the H_2_O buffer with D_2_O buffer. The pD of the D_2_O buffer was adjusted to 7.0 using the convention that the pH value of a D_2_O buffer read using a glass electrode is 0.4 pH unit lower than the pH reading of an H_2_O solution.^20^

### B. Raman measurements

Measurements of static visible RR spectra were conducted using the 532 nm line of a cw diode-pumped solid state laser (DPSS) (Cobolt, Samba 04-01) as the probe light. The sample solution was placed in a glass tube used as a spinning cell, and the 90° scattered Raman light was collected and focused onto the entrance slit of a spectrograph (HORIBA Jobin Yvon, iHR320) equipped with a CCD camera (Roper Scientific, PyLoN:400B_eXelon VISAR). The laser power was typically 1 mW at the sample point. The Raman shifts were calibrated using the Raman bands of cyclohexane, toluene, and acetone. The calibration error was within 1 cm^−1^.

Static UVRR spectra were measured using the 225 nm probe light obtained as the fourth harmonic of the output of a Ti:sapphire laser pumped by a Q-switched diode-pumped neodymium-doped yttrium lithium fluoride (Nd:YLF) laser (Photonics Industries, TU-L). The laser power was generally 0.5 mW at the sample point. The probe light was focused downward onto the sample cell at 45° and the 45° backscattered Raman light was collected and focused onto the entrance slit of a prefilter coupled to a spectrograph (HORIBA Jobin Yvon, iHR550) and detected with a CCD camera (Roper Scientific, SPEC-10:400B/LN-SN-U). The Raman shifts were calibrated using the Raman bands of cyclohexane, 2-propanol, and acetone. The calibration error was within 3 cm^−1^.

The experimental setup for picosecond time-resolved UVRR measurements has been described elsewhere.^14,18^ Briefly, the light source of our apparatus was a picosecond Ti:sapphire oscillator (Spectra-Physics, Tsunami pumped by Millennia-Vs) and amplifier (Spectra-Physics, Spitfire pumped by Evolution-15) system operated at 1 kHz. The wavelength and pulse energy were 796 nm and ∼800 *μ*J, respectively. The pump and probe pulses were generated by dividing the second harmonic of the laser output into two parts. The pump arm contained the optical parametric generation (OPG) and optical parametric amplification (OPA) device. The pump pulse was the output of the OPG/OPA system tuned to 565 nm with a width of ∼6 nm. In the probe arm, the 225 nm probe pulse was generated as the second harmonic of the first Stokes line of the CH_4_ Raman shifter and was introduced into an etalon to narrow the spectral width. The pump and probe pulses were collinearly focused onto a flowing thin-film of the sample solution by a plano–convex lens. The typical pulse energies were 5 *μ*J (pump) and 0.5 *μ*J (probe) at the sample point. The zero-delay time was precisely determined by measuring the intensity of the difference frequency generation between the pump and probe pulses. The cross correlation time between the pump and probe pulses was 3.4 ps. The Raman scattered light was collected and focused onto the entrance slit of a Czerny-Turner configured Littrow prism prefilter coupled to a 50-cm single spectrograph (SPEX, 500M) by two achromatic doublet lenses. The dispersed light was detected with a liquid nitrogen cooled CCD camera (Roper Scientific, SPEC-10:400B/LN). The Raman shifts were calibrated with the Raman bands of cyclohexane. The spectral dispersion was 3–3.5 cm^−1^/pixel on the CCD camera and the calibration error was within 10 cm^−1^.

Nanosecond-microsecond time-resolved RR measurements were performed using 475 nm probe pulses obtained as the second harmonic of the output of the Ti:sapphire laser pumped by a Q-switched diode-pumped Nd:YLF laser (Photonics Industries, TU-L). The pulse width of the probe laser was 30 ns. The pump pulses at 532 nm were generated with a Q-switched diode-pumped Nd:YAG laser (Megaopto, LR-SHG). The pulse width was 20 ns. The repetition rate of both lasers was 1 kHz. The pulse energies of the probe and pump pulses were typically 1.6 and 65 *μ*J at the sample point, respectively. The 45° backscattered Raman light was collected and focused onto the entrance slit of the prefilter coupled to a spectrograph (HORIBA Jobin Yvon, iHR550) and detected with a CCD camera (Roper Scientific, SPEC-10:400B/LN-SN-U). The Raman shifts were calibrated using the Raman bands of cyclohexane, 2-propanol, and acetone. The calibration error was within 3 cm^−1^.

The anion-depleted form provided a band at 406 nm in addition to the band at 600 nm, as shown in Fig. S2A. The 475 nm probe condition resulted in an additional spectral contribution of the retinal chromophore with an absorption band at 406 nm in the raw spectra. The spectral contribution of the 406 nm species has been subtracted from the raw spectra, as described in the supplementary material (Fig. S2 and related text).

## III. RESULTS

### A. Steady-state RR spectra

**FIG. 1.**
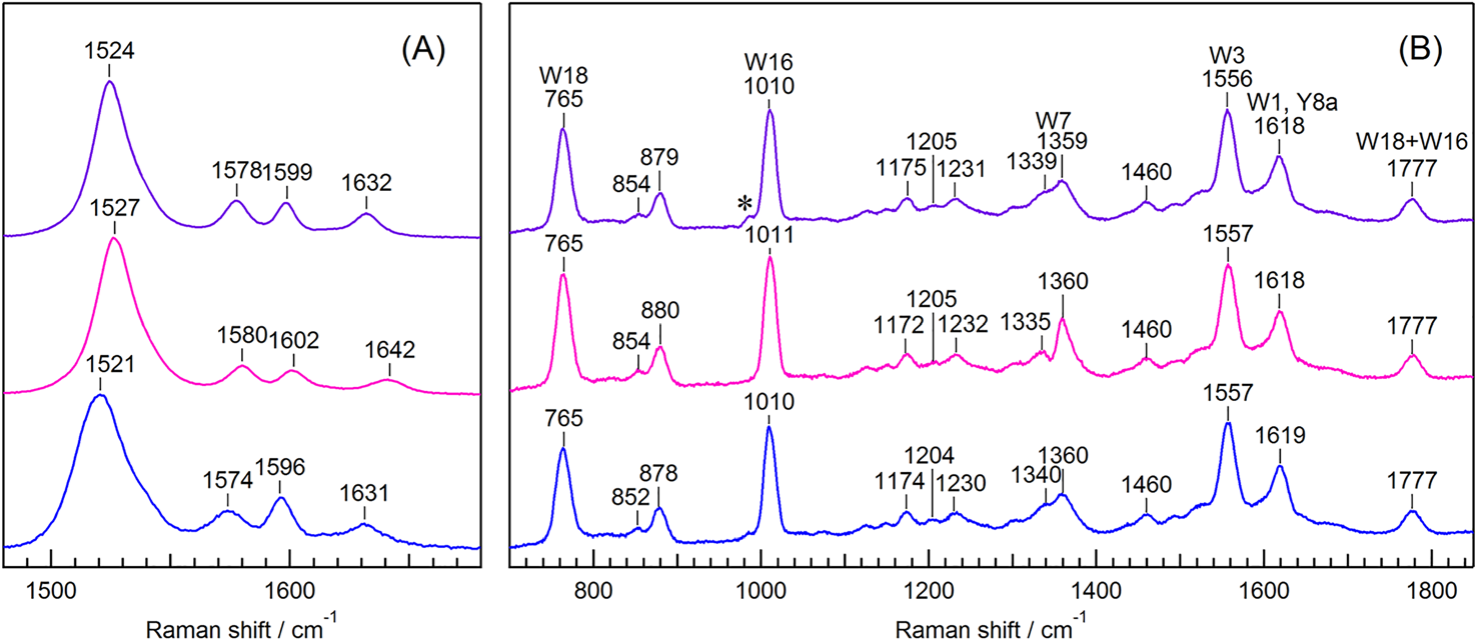
Visible RR spectra excited at 532 nm (a) and UVRR spectra excited at 225 nm (b) of the unphotolyzed state of NpHR. Purple, magenta, and blue traces are spectra of the chloride-bound, formate-bound, and anion-depleted forms, respectively. The asterisk in panel B indicates the band due to the sulfate ion added as an internal intensity standard.

Figure 1(a) displays visible RR spectra of NpHR in the ethylenic C=C stretch and C=N stretch region excited at 532 nm. The most intense in-phase C=C stretch band was observed at 1524 cm^−1^ for the chloride-bound form. The corresponding bands were observed at 1527 and 1521 cm^−1^ for the formate-bound and anion-depleted forms, respectively. The observed difference in the C=C stretching frequency implies that the bound anion directly and/or indirectly affects the retinal π-conjugation. This interaction is also evident in the difference in the absorption maximum wavelengths of the three forms. The absorption maximum wavelengths were 578, 564, and 600 nm for the chloride-bound, formate-bound, and anion-depleted forms, respectively.^10,12^ The C=C stretching frequency and the absorption maximum wavelength observed for NpHR are consistent with an inverse linear correlation with the absorption maximum wavelengths, as reported for the RR spectra of the retinal chromophore of HsHR^8^ and other rhodopsins.^21^

The C=N stretch band at the protonated Schiff base appeared at 1632 cm^−1^ for the chloride-bound form, 1642 cm^−1^ for the formate-bound form, and at 1631 cm^−1^ for the anion-depleted form. In D_2_O, the C=N stretching frequency shifted to 1622, 1626, and 1614 cm^−1^ for the chloride-bound, formate-bound, and anion-depleted forms, respectively (Fig. S3 in the supplementary material). These bands result from the C=N stretching mode coupled with the in-plane N–H rocking mode. The C=N stretching and the N−D rocking modes are decoupled in D_2_O. The observed deuteration shifts indicate that the Schiff base is protonated in all three forms of NpHR. Furthermore, the decoupled C=N stretching frequencies observed in D_2_O show the intrinsic frequencies of the C=N stretching mode. The observed C=N stretching frequencies in D_2_O showed a linear correlation with the absorption maximum wavelengths, as observed for the C=C stretching frequencies, implying that the C_15_=N moiety is involved in π-conjugation. The magnitude of the deuteration shift provides a good measure of the strength of the hydrogen bond formed by the protonated Schiff base.^22^ The magnitudes of the deuteration shifts were 10, 16, and 17 cm^−1^ for the chloride-bound, formate-bound, and anion-depleted forms, respectively. The hydrogen bond for the chloride-bound form is very weak, as we reported previously.^13^ The observed deuteration shifts for the formate-bound and anion-depleted forms are large and comparable to those for other proton pumps^23–27^ and sodium ion pumps,^28,29^ indicating that the hydrogen bond is strong for both forms. An alternative mechanism was proposed to account for nature of the C=N stretching mode.^30,31^ In the case of protonated Schiff base of NpHR, where the proton is closer to the nitrogen atom of the Schiff base than to a counterion, the stretch-rock coupling mechanism makes a maximal contribution.

Figure 1(b) shows UVRR spectra of the unphotolyzed state excited at 225 nm. The spectrum contains all the bands arising from the 8 tryptophan and 11 tyrosine residues in NpHR because the Raman bands of the tryptophan and tyrosine residues are resonantly enhanced under the present probe light conditions. We adopted the mode assignments of Harada and Takeuchi^32^ and Asher *et al.*^33^ The observed band frequencies of the three bound forms were similar, indicating that the protein structure changes little upon anion binding. The intensities of the 765 and 1010 cm^−1^ bands were highest in the formate-bound form, followed by the chloride-bound and then the anion-depleted forms (Fig. S4), and were assigned to the W18 and W16 modes, respectively. It is known that the intensities of the W18 and W16 bands increase with increasing hydrophobicity.^34^ The intensity differences indicate that the environment of the tryptophan residues in the formate-bound form is likely more hydrophobic than the those in the other forms.

### B. Picosecond time-resolved UVRR spectra

**FIG. 2.**
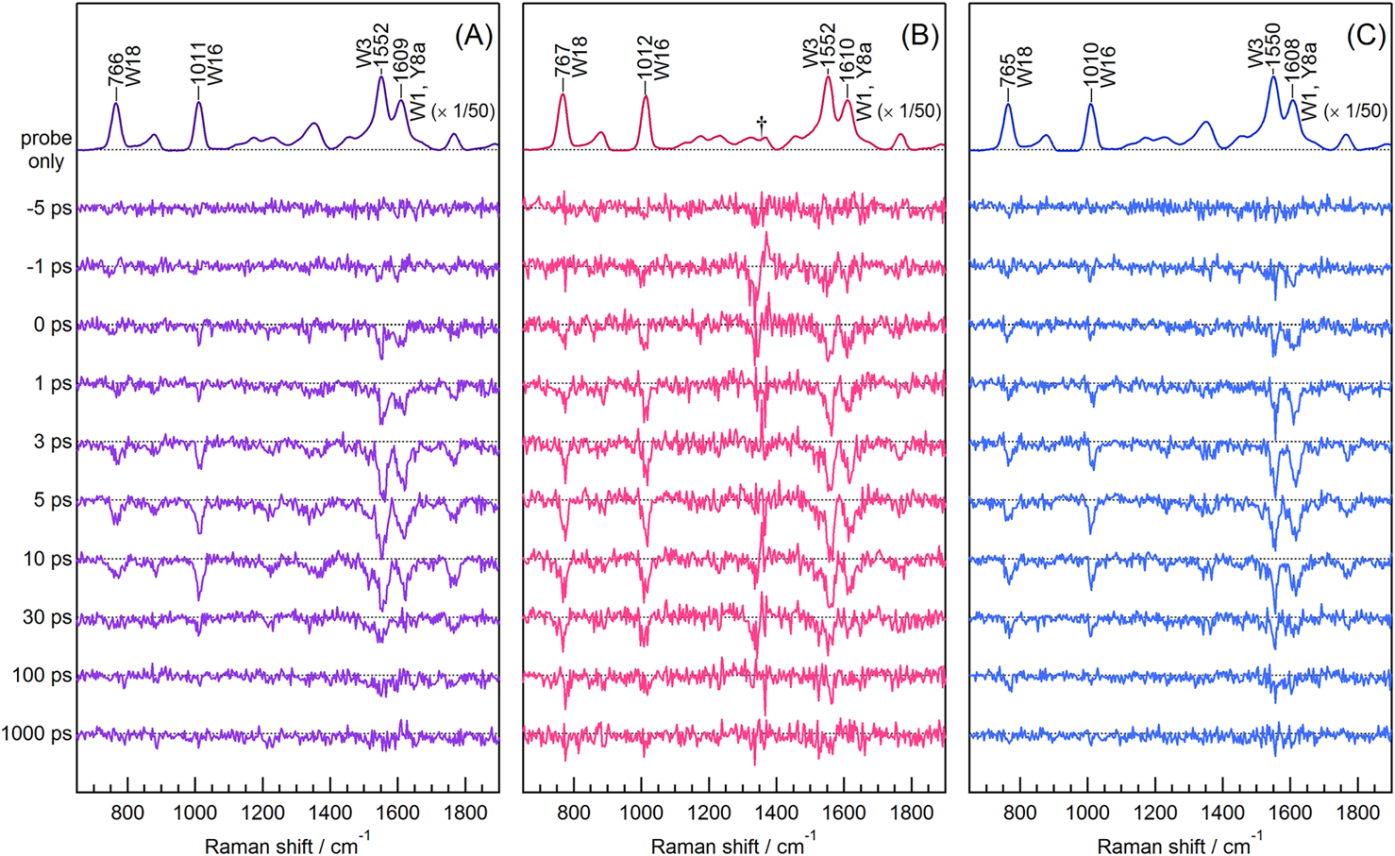
Picosecond time-resolved UVRR spectra of NpHR. (a) Chloride-bound form (150 mM NaCl), (b) formate-bound form (1 M NaHCOO), and (c) anion-depleted form (50 mM Na_2_SO_4_). The probe and pump wavelengths were 225 and 565 nm, respectively. The top trace in each panel is the spectrum without pump pulse irradiation divided by a factor of 50 and represents the UVRR spectrum of the unphotolyzed state. The spectrum of the buffer has been subtracted. The other spectra are time-resolved difference spectra generated by subtracting the spectral contribution of the unphotolyzed state at each delay time. The dagger in panel B indicates the band position of the formate ion which severely influences the spectral shape of the difference spectra. The accumulation times for obtaining each spectrum of the chloride-bound, formate-bound, and anion-depleted forms were 131, 136, and 146 min, respectively.

Figure 2(a) shows picosecond time-resolved UVRR spectra of chloride-bound NpHR probed at 225 nm. The top trace is the UVRR spectrum of the unphotolyzed state measured using only the picosecond probe pulses. The other traces in Fig. 2(a) are time-resolved difference spectra of the UVRR spectra with and without pump pulse irradiation. These are one-to-one differences between the spectra after correction of self-absorption effect using Raman intensity of a sulfate band at 980 cm^−1^. The time-resolved spectra were measured in the time range from −5 to 1000 ps. Following photoexcitation, negative bands appeared at the positions of the bands of the tryptophan and tyrosine residues. These negative bands indicate bleaching of the Raman intensity due to changes in the protein structure in response to photoisomerization of the chromophore, which occurs within 1 ps.^35^ At 3 ps, the amplitudes of the negative signals were as large as 1.5% compared to the Raman intensities of the unphotolyzed state. The negative signals decayed in 100 ps. The spectral contribution of the retinal chromophore is negligible in the time-resolved difference spectra because of the low Raman cross section of retinal under the present probe condition.^14^ Figures 2(b) and 2(c) display the picosecond time-resolved UVRR spectra of formate-bound and anion-depleted NpHR, respectively. The dagger in panel B indicates the band position of the formate ion which severely influences the spectral shape of the difference spectra. Similar to the spectra of the chloride-bound form, the negative bands increased up to 3 ps in the time-resolved difference spectra at the positions of the bands of the tryptophan and tyrosine residues and decayed between 5 and 100 ps. Intensity changes in the tryptophan bands on the picosecond time scale were also observed for other microbial rhodopsins, such as BR from *Halobacterium sarinarum* (HsBR),^14^ sensory rhodopsin II from *Natronomonas pharaonis* (NpSRII),^15^ sensory rhodopsin I from *Salinibacter ruber* (SrSRI),^17^ and *Anabaena* sensory rhodopsin (ASR),^16^ as well as visual pigment rhodopsin.^36^ Intensity decay of the negative difference bands with a time constant of a few tens of picoseconds is common among these rhodopsins.

**FIG. 3.**
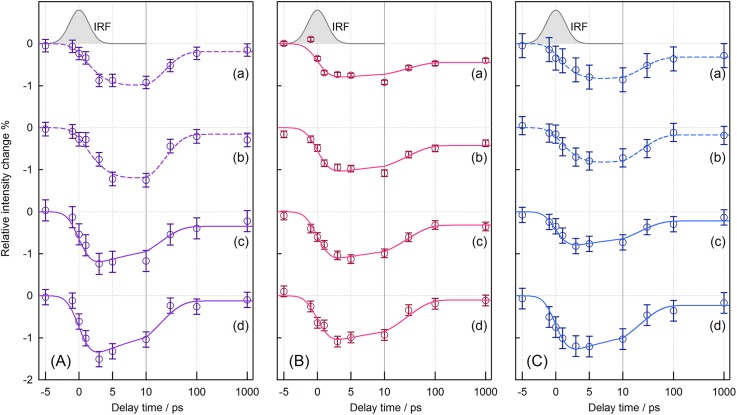
Temporal intensity changes of the UVRR bands of NpHR. (panel a) Chloride-bound form, (panel b) formate-bound form, and (panel c) anion-depleted form. The open circles represent the intensity changes for (trace a) the W18 band, (trace b) the W16 band, (trace c) the W3 band, and (trace d) the overlap of the W1 and Y8a bands, measured at each delay time relative to the intensity of the spectrum of the unphotolyzed state. Curves are the best-fits obtained using the function [*A*_1_ × exp(−*t*/*τ*_fast_) + *A*_2_ × exp(−*t*/*τ*_slow_) + *A*_3_] convoluted with the instrument response function. The solid curves were obtained using an amplitude of zero for *A*_1_. An instrument response function (IRF) of the time-resolved UVRR apparatus is shown at the top of each panel for comparison.

Temporal intensity changes of the UVRR bands are shown in Fig. 3. Figure 3(a) represents the intensity changes of the four intense bands (W18, W16, W3, and the overlap of W1 and Y8a) for the chloride-bound form. The appearance of the negative signal for the W18 and W16 bands was delayed with respect to the instrument response [traces (a) and (b)], whereas the W3 band and the overlap of the W1 and Y8a bands instantaneously bleached [traces (c) and (d)]. The negative signals of the four bands decayed with a similar time constant. To reproduce the observed temporal intensity changes, we used the fitting function [*A*_1_ × exp(−*t*/*τ*_1_) + *A*_2_ × exp(−*t*/*τ*_2_) + *A*_3_] convoluted with an instrument response function (IRF). The parameters *τ*_1_ and *τ*_2_ indicate the time constants of the delayed bleach and intensity recovery, respectively. The amplitude parameter *A*_1_ was regarded zero for the intensity changes in the W3 band and the overlap of the W1 and Y8a bands. Using the global parameters of *τ*_1_ and *τ*_2_ for the observed intensity changes, we calculated the time constants of the delayed bleach and intensity recovery to be 2.8 ± 1.1 and 19.2 ± 3.8 ps, respectively. The different temporal behaviors of the intensity changes of the observed tryptophan bands imply that the observed spectral changes are attributable to structural changes of at least two residues.

Figure 3(b) displays the temporal behaviors of the UVRR bands of the formate-bound form and shows that the UVRR intensities instantaneously decreased, in contrast to the intensity changes observed for the W18 and W16 bands of the chloride-bound form. The time constant of the intensity recovery, *τ*_2_, for the formate-bound form was calculated to be 29.0 ± 6.0 ps. Figure 3(c) shows the temporal changes in the UVRR bands of anion-depleted NpHR. As with the spectra of the chloride-bound form, the temporal behaviors of the W18 and W16 bands were different from those of the W3 band and the overlap of the W1 and Y8a bands. The time constant of the delayed bleach, *τ*_1_, for the W18 and W16 bands was 4.1 ± 1.9 ps, while that of intensity recovery, *τ*_2_, was 21.4 ± 3.4 ps. These time constants are comparable to those observed for the chloride-bound form. Overlays of the temporal intensity changes for the three forms of NpHR are shown in Fig. S5.

To identify the tryptophan residue(s) responsible for the spectral changes of the UVRR bands, we attempted to obtain NpHR mutants in which each tryptophan residue was substituted with phenylalanine. However, the preparation of mutants, in which Trp121, Trp177, Trp179, Trp198, Trp222, and Trp246 were replaced with phenylalanine, was unsuccessful because of low protein expression and low affinity of the proteins for the retinal chromophore. In contrast, sufficient amounts of the W127F and W229F NpHR mutants were obtained to measure their spectra. We found that both the absorption band of the retinal chromophore and the affinity of the protein for chloride ion were affected by substitution of the tryptophan residue (Fig. S7) because the substitution alters the protein structure around the retinal chromophore. The time-resolved UVRR spectra and the temporal behaviors of the tryptophan bands were similar between the two mutants and the wild type protein (Fig. S8). However, a difference was seen in the initial bleach of the W18 band of the W127 mutant: Namely, the W18 band intensity instantaneously decreased for the W127 mutant while those for the wild type and W229F mutant did not. Thus, Trp127 contributes to the UVRR intensity change of the W18 band observed in Fig. 2.

### C. Nanosecond-microsecond time-resolved visible RR spectra

**FIG. 4.**
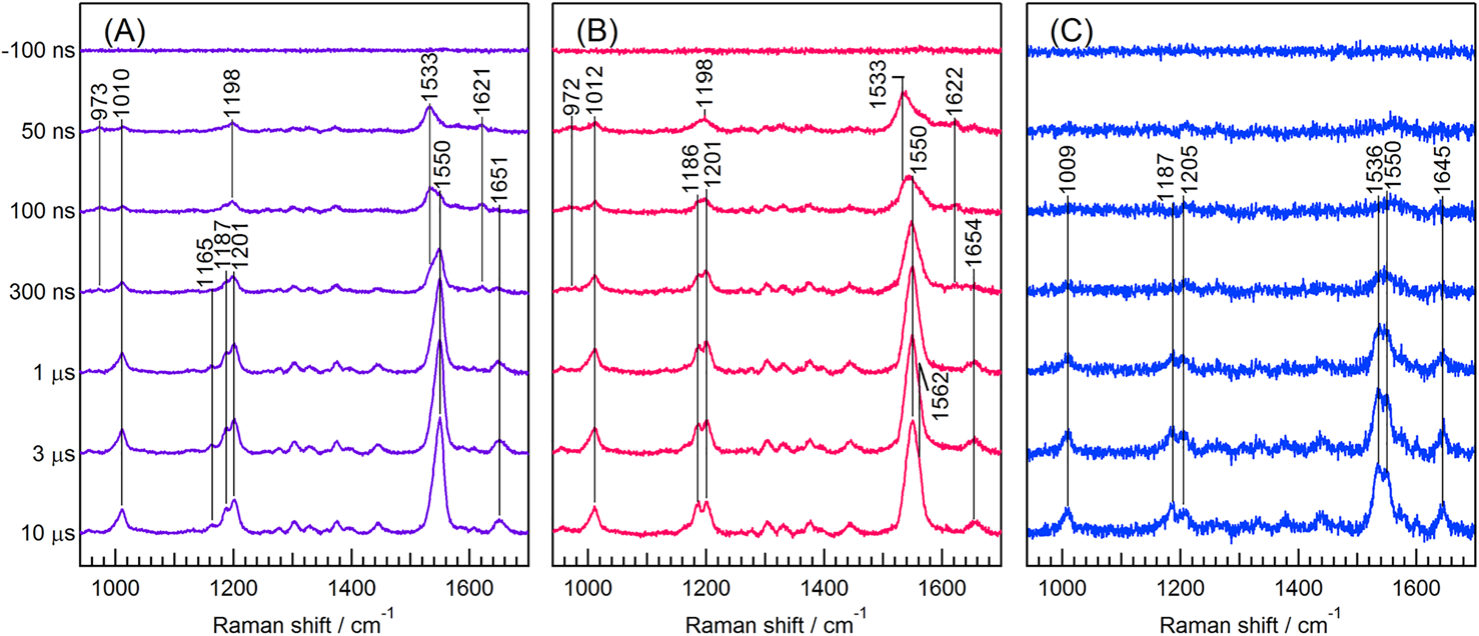
Nanosecond-microsecond time-resolved visible RR spectra of the retinal chromophore in NpHR. (a) Chloride-bound form (150 mM NaCl and 283 mM Na_2_SO_4_), (b) formate-bound form (1 M NaHCOO), and (c) anion-depleted form (333 mM Na_2_SO_4_). The probe and pump wavelengths were 475 and 532 nm, respectively. The spectrum of the buffer has been subtracted. The other spectra are time-resolved difference spectra generated by subtracting the spectral contribution of the unphotolyzed state at each delay time. The accumulation time for obtaining each spectrum was 10 min.

Figure 4(a) shows the time-resolved difference visible RR spectra of chloride-bound NpHR obtained by subtracting the spectral component of the unphotolyzed state at each delay time. As reported previously,^13^ spectral contributions of the retinal chromophore in the K and L intermediates are involved in the time-resolved spectra in the time window from 50 ns to 10 *μ*s. The bands at 973, 1010, 1198, 1533, and 1621 cm^−1^ were observed at 50 ns and are assigned to the K intermediate. The bands at 1533 and 1621 cm^−1^ decayed in 300 ns. After 100 ns, new bands appeared at 1165, 1187, 1201, 1550, and 1651 cm^−1^ and are due to the L intermediate.

Figure 4(b) displays the time-resolved RR spectra of formate-bound NpHR. Similar to the chloride-bound form, transient Raman bands due to the K intermediate were observed after photoexcitation at 972, 1012, 1198, 1533, and 1622 cm^−1^. The bands due to the K intermediate at 1533 and 1622 cm^−1^ decreased in intensity whereas the bands at 1186, 1201, 1550, and 1654 cm^−1^ increased in intensity and are attributed to the L intermediate. Finally, a shoulder band appeared at 1562 cm^−1^ after 3 *μ*s and is attributed to the C=C stretch band of the M intermediate, previously reported for the formate-bound form.^12^

Figure 4(c) shows the time-resolved difference RR spectra of anion-depleted NpHR. The spectral contribution of the retinal chromophore with an absorption band at 406 nm has been subtracted. After 100 ns, Raman bands appeared at 1009, 1187, 1205, 1536, 1550, and 1645 cm^−1^, and their intensities increased up to 10 *μ*s. The features observed in the present time-resolved spectra were very similar to the RR spectrum of the L intermediate of anion-depleted NpHR excited at a 514 nm using a single-beam time-resolved method.^37^ Transient absorption spectroscopy of anion-depleted NpHR showed that the L intermediate appears up to 10 *μ*s, and then simply decays to the ground state.^10^ Thus, the transient RR bands observed in Fig. 4(c) are solely attributed to the L intermediate of anion-depleted NpHR. No bands due to the K intermediate were observed because of low resonant enhancement. A characteristic doublet feature appeared at 1536 and 1550 cm^−1^ for the C=C stretching mode, previously observed in the RR spectrum of the L intermediate of HsBR.^38,39^ Spectral differences between the L intermediates of the anion-depleted and anion-bound forms indicate that the difference in the chromophore in the L intermediate is associated with the difference in the subsequent reaction pathway in the photocycles between the anion-depleted and anion-bound forms.

Next, we compared the spectral features of the chloride- and formate-bound forms. The transient RR bands of the K intermediate were observed in the spectrum at 50 ns for both forms and the spectral patterns were very similar. For example, the C=C and C=N stretching frequencies were nearly identical for the two forms. This suggests that the structures of the retinal chromophore in the K intermediate are similar between the two anion-bound forms, implying that the bound anion has little effect on the chromophore structure in the K intermediate of NpHR. This contrasts with the spectra of the unphotolyzed state, shown in Fig. 1(a).

For the chloride- and formate-bound forms, the C=N stretching frequencies in the K intermediates were lower than those in the unphotolyzed state, showing that the hydrogen bond is weakened upon formation of the K intermediate. The C=N stretching frequencies in the K intermediates of NpHR were also lower than those in the K intermediates of other ion pumps.^29,40^

The spectra of the chloride- and formate-bound forms at 1 *μ*s show that the observed RR bands are dominantly due to the L intermediate. The C=N stretch band was observed at 1651 cm^−1^ for the chloride-bound form, whereas it appeared at 1654 cm^−1^ for the formate-bound form. This frequency difference indicates that the hydrogen bond in the Schiff base of the formate-bound form is stronger than that of the chloride-bound form. The strong C=C stretch band observed at 1550 cm^−1^ in the spectra of both forms indicates that the π-conjugation system of the polyene chain is insensitive to the anion bound to the protein.

**FIG. 5.**
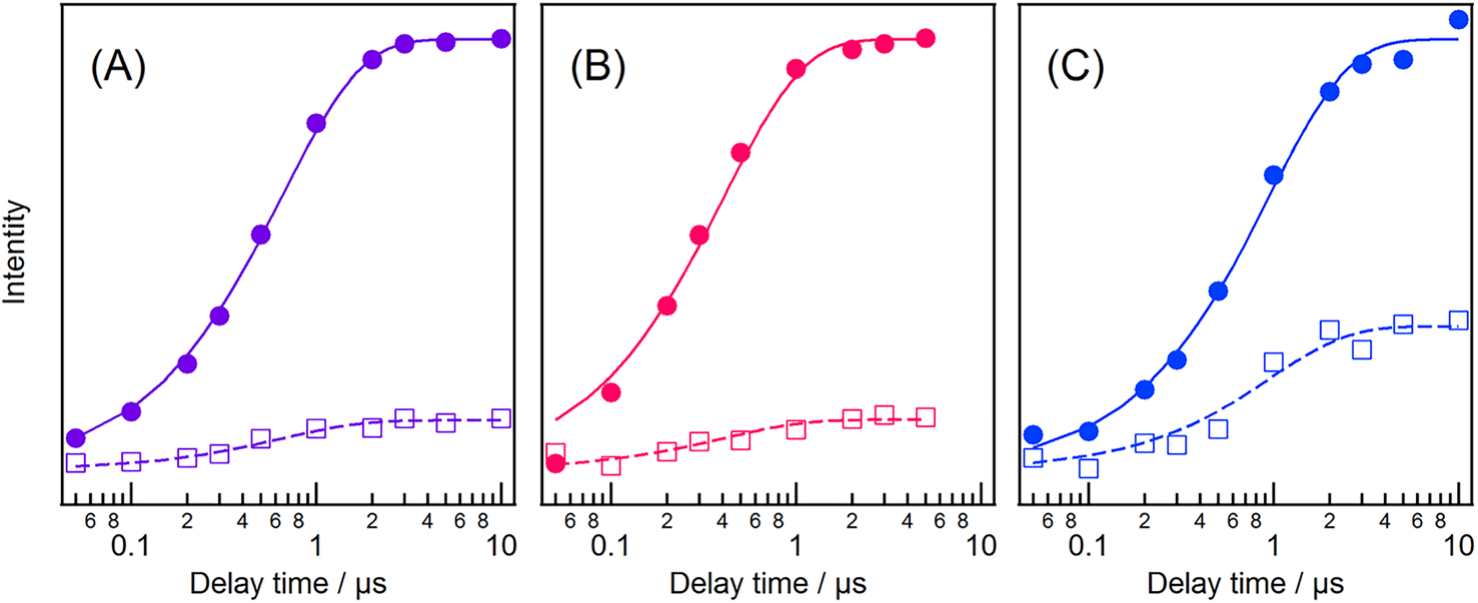
Temporal intensity changes of the RR bands of the L intermediate. (a) Chloride-bound form, (b) formate-bound form, and (c) anion-depleted form. Closed circles and open squares represent intensity changes of the C=C stretch and the C=N stretch bands, respectively. Curves indicate the best-fit to a single exponential function.

Figure 5 shows the temporal intensity changes in the C=C and C=N stretch bands of the L intermediate. These intensity increases are indicative of the formation of the L intermediate. The temporal intensity changes in these bands were well reproduced by a single exponential function. The time constant of the intensity increase for the chloride-bound form was 0.65 ± 0.01 *μ*s and is similar to the value reported previously.^13^ The time constant was reduced to 0.41 ± 0.02 *μ*s for the formate-bound form and increased to 0.94 ± 0.05 *μ*s for the anion-depleted form, indicating that the bound anion affects the energy barrier for the formation of the L intermediate in NpHR. Overlays of the temporal intensity changes for the three forms of NpHR are shown in Fig. S6.

## IV. DISCUSSION

### A. Protein structure around the retinal chromophore in the unphotolyzed state

The spectral features of the observed visible RR bands of the retinal chromophore in the unphotolyzed state of NpHR depend on the anion bound to the protein as summarized in Table I. The C=C stretching frequencies were 1524, 1527, and 1521 cm^−1^, whereas the C=N stretching frequencies in D_2_O (the intrinsic C=N stretching frequency) were 1622, 1626, and 1614 cm^−1^ for the chloride-bound, formate-bound, and anion-depleted forms, respectively, as shown in Figs. 1(a) and S3. These frequencies exhibited an inverse correlation with the respective absorption maximum wavelengths observed at 578, 564, and 600 nm. Taken together, these results show that the bound anion affects the π-conjugation of the retinal chromophore. The UVRR spectra of the three forms were similar to each other [Fig. 1(b)] except for the intensity differences of the Trp residues, suggesting that the protein structure is not greatly affected by the anion bound to the protein.

**TABLE I.** Vibrational assignments of visible RR bands of the retinal chromophore. Numbers in parentheses indicate the C=N stretching frequencies in D_2_O.

	Vibrational frequencies (cm^−1^)		
Assignments	Chloride-bound NpHR	Formate-bound NpHR	Anion-depleted NpHR
	Unphotolyzed state		
C=C stretch	1524	1527	1521
C=N stretch	1632 (1622)	1642 (1626)	1631 (1614)
	K intermediate		
C=C stretch	1533	1533	nd
C=N stretch	1621	1622	nd
	L intermediate		
C=C stretch	1550	1550	1536
			1550
C=N stretch	1651	1654	1645

**FIG. 6.**
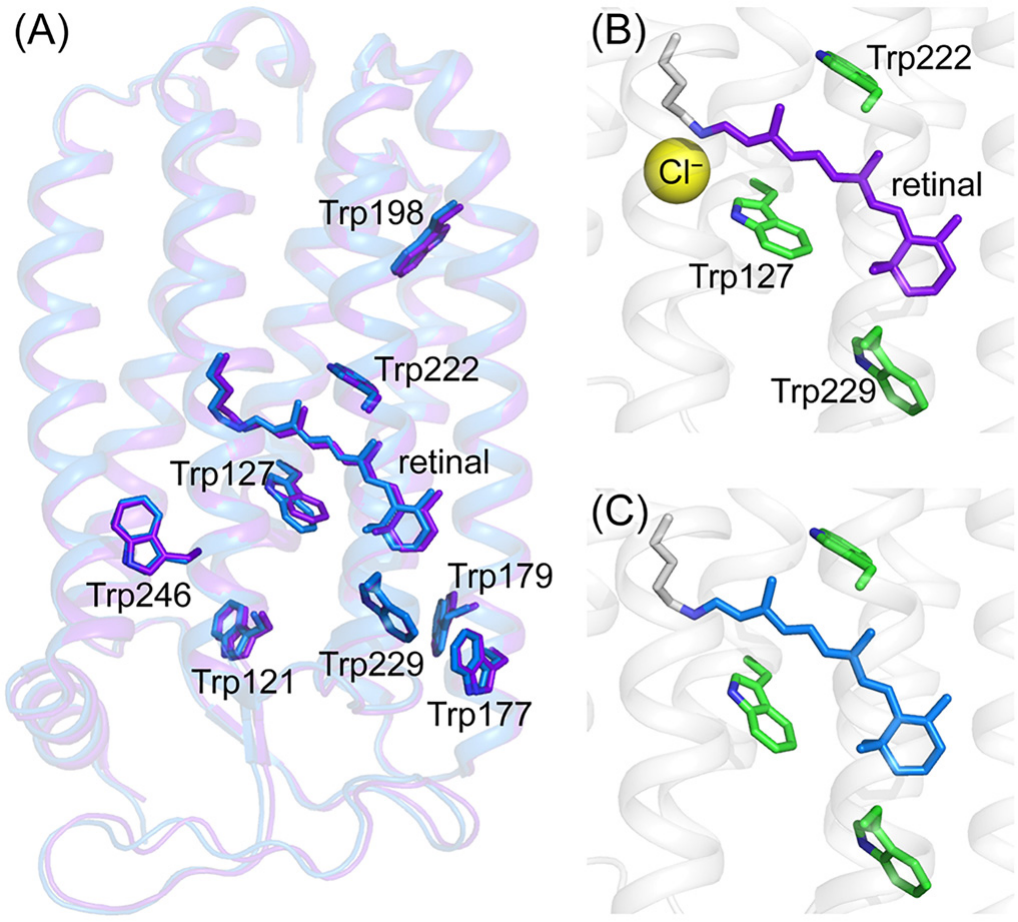
Comparison of the crystallographic structures of NpHR. (a) Overall structures for the chloride-bound form (purple, PDB ID: 3A7K) and the anion-depleted form (blue, PDB ID: 3QBG). All tryptophan residues in NpHR are depicted in panel (a). The structure around the retinal chromophore of the chloride-bound form (b) and the anion-depleted form (c). The chloride bound to the protein is shown as a yellow sphere in panel (b). The three tryptophan residues (Trp127, Trp222, and Trp229) whose side chains are located within 5 Å of the retinal chromophore are shown.

**FIG. 7.**
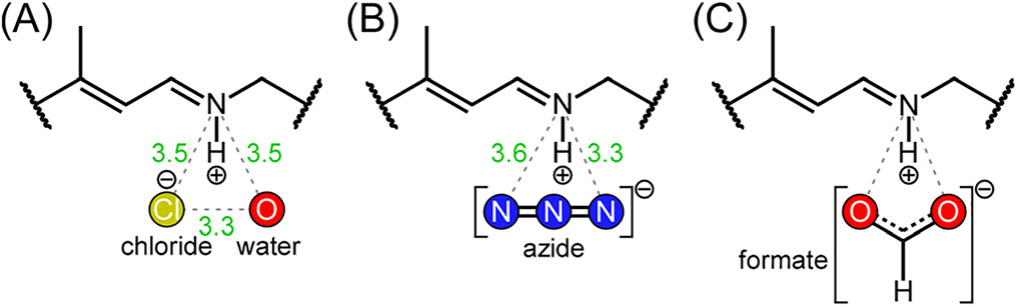
Structures of the Schiff base region in NpHR. Structures for the chloride-bound (a) and the azide-bound forms (b) are drawn on the basis of the crystallographic data of which PDB IDs are 3A7K and 3ABW, respectively. Numbers show distances (in angstrom) between atoms connected by dotted lines. Shown in (c) is a putative structure for the formate-bound form, which is drawn on the analogy of the structure of the azide-bound form in (b).

The crystallographic structures of the unphotolyzed states of the chloride-bound (PDB ID: 3A7K^6^) and anion-depleted (PDB ID: 3QBG^41^) forms have been reported and are shown in Fig. 6. Although the crystallographic structure of the formate-bound form is not available, the structures of an azide-bound form (PDB ID: 3ABW^7^) and a nitrate-bound form (PDB ID: 3QBL) were reported for NpHR containing the polyatomic anion. The overall structures of these forms are very similar to each other. The similarity between the present UVRR spectra of the chloride-bound and anion-depleted forms is consistent with the similarity between the crystallographic structures of the two forms. Based on the structure of the chloride-bound form, the chloride is located near the Schiff base of the chromophore. Figure 7(a) shows the structure of the region of the protonated Schiff base in the chloride-bound form, which was drawn based on the crystallographic data (PDB ID: 3A7K). The binding site for the polyatomic anion is similar to that for the chloride ion. Thus, it is highly likely that the binding site of the formate ion is located near the protonated Schiff base of the retinal chromophore. The putative structure of the formate-bound form is shown in Fig. 7(c), which was postulated on the basis of the crystallographic structures of the azide-bound form (PDB ID: 3ABW) shown in Fig. 7(b). Since the negative charge of the anion located near the retinal chromophore results in electrostatic interaction with the polyene chain of the chromophore, bond alternation in the π-conjugation of the polyene chain is enhanced upon anion binding, leading to the observed blue shift of the maximum wavelength and the upshift of the C=C/C=N stretching frequencies.

The hydrogen bond strength of the protonated Schiff base can be monitored by the C=N stretch band. We previously reported that both the C=N stretching frequency and its deuteration shift are insensitive to the bound halide ion in the unphotolyzed state.^13^ In contrast, in the present study we found that the C=N stretching frequency and its deuteration shift for the formate-bound form were distinct from those for the chloride-bound form. This observed spectral difference means that the hydrogen bond of the protonated Schiff base is strengthened upon formate binding. The Schiff base in the chloride-bound form was reported to have a p*K*_a_ value of 10 and it decreased to 8.5 in the formate-bound form, resulting in an anion effect on the HR photocycle.^12^ The p*K*_a_ value of the formate ion is 4^42^ and is much larger than those of chloride, bromide, and iodide ions (−7 to −10 at 300 K),^43^ implying that the proton affinity of formate is much higher than those of halide ions. The basicity of the anions bound to the protein is associated with the strength of the hydrogen bond formed by the protonated Schiff base.

### B. Primary protein response to the photoreaction of the retinal chromophore

Intensity decreases were observed for the UVRR bands on the picosecond time scale following photoexcitation of the chromophore (Fig. 2). Using 225 nm probe light, Raman bands of the tryptophan residues were predominantly observed in the UVRR spectra. The RR intensity of tryptophan is known to be sensitive to the protein environment around the residue.^34,44^ At the red edge of the Raman excitation profile, the Raman cross section decreases due to the blue shift of the absorption band at 220 nm with increasing the hydrogen bond strength of the N–H group in the indole ring and the polarity around the residue.^34^ Changes in the excitonic coupling between the retinal chromophore and its nearby tryptophan residue(s) upon the photoexcitation would change the absorption spectrum of the tryptophan residue, as reported for BR.^45^ These changes are responsible for the observed intensity changes of the tryptophan residues in NpHR on the picosecond time scale.

For the chloride-bound form, the temporal intensity changes of the W18 and W16 bands were replicated with a double-exponential function, whereas those of the W3 band and the overlap of the W1 and Y8a bands can be represented as a single-exponential function, as shown in Fig. 3(a). These results indicate that the observed spectral changes consist of at least two spectral components: One shows intensity bleaching within the instrumental response (instantaneous bleach component) and the other exhibits an intensity decrease with a time constant of 3 ps (delayed bleach component). The observed spectral changes of the Trp residues are due to the combination of the contributions from two types of tryptophan residues: Those that respond instantaneously to photoexcitation, and those that respond with a delay to photoexcitation. The response of the former is due to the formation of the electronic excited state of the retinal chromophore. The response of the latter is consistent with the reported experimental observation that the isomerization process to generate the electronic ground-state 13-*cis* product occurs with a time constant of about 1 ps^35^ and that the intensity of the amide II band changes with a time constant of 2 ps in time-resolved IR spectra of HsHR.^46^

After 5 ps, the band intensities recovered with a time constant of 20 ps. Time-resolved IR spectroscopy of HsHR showed that the bleach signal of the amide II band recovered within 15 ps due to protein relaxation after chromophore isomerization.^46^ Thus, the intensity recoveries observed in the present UVRR spectra of NpHR are due to structural rearrangement of the protein moiety following the primary structural changes discussed in the preceding paragraph.

**FIG. 8.**
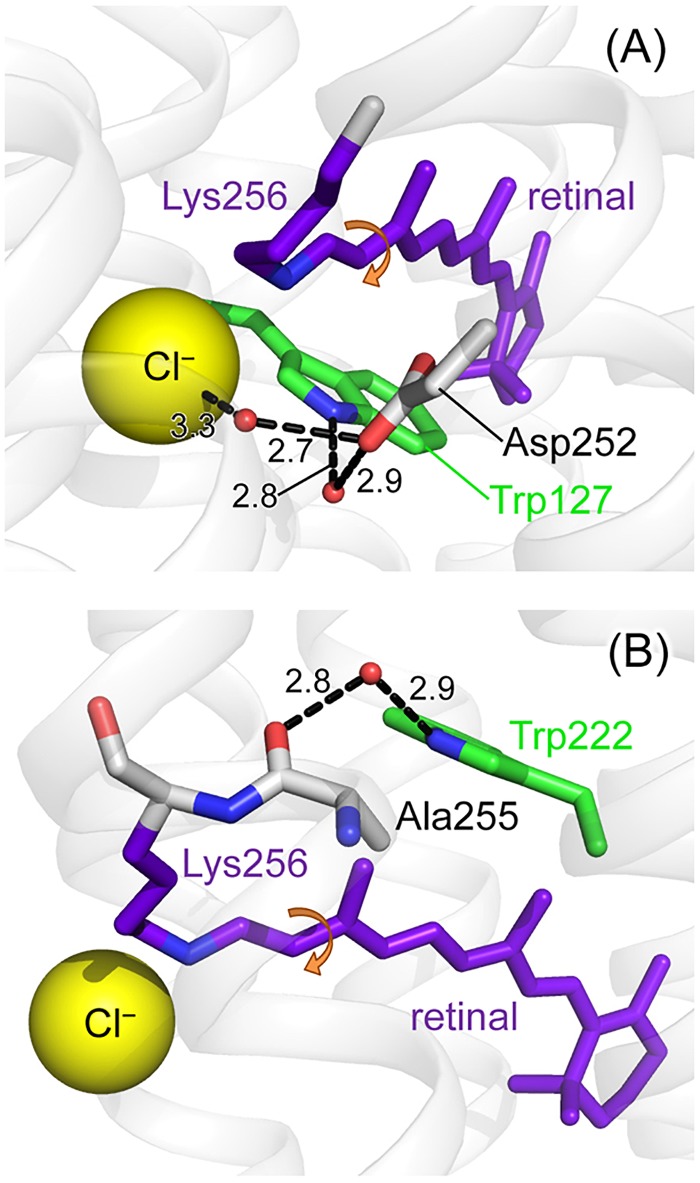
Crystallographic structure of chloride-bound NpHR in the unphotolyzed state (PDB ID: 3A7K). The protein structure around Trp127 (a) and Trp222 (b). Numbers in the figure represent distances in angstrom between atoms at both ends of the dashed lines. Round arrows indicate the isomerized position of the retinal chromophore.

We attempted to identify the tryptophan residue contributing to the observed spectral changes in the time-resolved UVRR spectra. There are eight tryptophan residues in an NpHR molecule, as shown in Fig. 6(a), of which four (Trp121, Trp177, Trp179, and Trp246) are on the protein surface. Trp198 is an interior residue but is very far (over 10 Å) from the retinal chromophore. Changes in the protein moiety in response to the photoreaction of the chromophore are very limited on the early picosecond time scale and thus, the spectral contributions of these tryptophan residues would be negligible in the observed spectra. In contrast, the side chains of Trp127, Trp222, and Trp229 are located within 5 Å of the retinal chromophore. Trp127 and Trp222 are located near the protonated Schiff base of the chromophore, whereas Trp229 is close to the β-ionone ring of retinal. Consequently, Trp127 and Trp222 are the most probable candidates responsible for the observed spectral changes on the picosecond time scale because the retinal chromophore isomerizes at the C_13_=C_14_ bond near the Schiff base, as displayed in Fig. 8.

Based on the time-resolved UVRR spectra of the W127F and W229F mutants (Fig. S8), the spectral contribution of Trp127 is probably involved in the delayed bleaching of the W18 band. The other interior tryptophan residue close to the retinal chromophore, Trp222, is likely involved in instantaneous bleaching. Trp127 is located very close to the protonated Schiff base and the chloride ion bound to the protein. Its side chain N–H group is linked to the bound chloride ion through a hydrogen bond network involving water molecules and Asp252 [Fig. 8(a)]. Trp222 and Trp127 are on opposite sides of the chromophore. The N–H group in the Trp222 side chain is hydrogen-bonded to the carbonyl oxygen of the main chain in Ala255, which neighbors Lys256 bound to the retinal chromophore via a water molecule [Fig. 8(b)]. Structural changes propagate through these linkages following chromophore isomerization.

We examined the anion dependence of intensity bleaching in the time-resolved UVRR spectra of NpHR (Figs. 2 and 3). Compared to the spectral change of the chloride-bound form, no delayed bleach component was observed for the W18 and W16 bands in the spectra of formate-bound NpHR. As mentioned above, delayed bleaching is likely due to structural change in the protein moiety around Trp127 close to the anion binding site [Fig. 8(b)]. The C=N stretch band showed that the protonated Schiff base forms a stronger hydrogen bond in the formate-bound form than in the chloride-bound form, suggesting that the formate ion interacts with the chromophore more strongly than the chloride ion. Therefore, the structural changes to Trp127 can propagate faster because of the resultant tight coupling between the formate ion and the retinal chromophore.

Intensity recovery at 20–30 ps resulting from structural rearrangement was observed for all three forms of NpHR. Spectral changes with similar time constants were observed in the time-resolved UVRR spectra of microbial rhodopsins, such as HsBR,^14^ NpSRII,^15^ SrSRI,^17^ and ASR,^16^ as well as visual pigment rhodopsin,^36^ indicating that the rates of rearrangement of the protein moiety are insensitive to function. It should also be noted that insensitivity of the rate of protein rearrangement upon ion binding was observed in SrSRI.^17^ Moreover, it was observed that the rearrangement rates of ASR were insensitive to the direction of isomerization (*trans*-to-*cis* vs *cis*-to-*trans*).^16^ These results suggest that the primary structural response of the protein moiety to chromophore isomerization is very similar irrespective of both the bound ion and the mode of structural change of the chromophore in microbial rhodopsins.

### C. Structural changes in the retinal chromophore following photoisomerization

We observed differences in the chromophore structures of the intermediates appearing on the nanosecond to microsecond timescales between the chloride- and formate-bound forms. The C=N and C=C stretching frequencies for the chloride- and formate-bound forms of the K intermediate were similar, in sharp contrast to the marked anion dependence observed for the unphotolyzed state. The C=N stretching frequency indicated that the hydrogen bond of the protonated Schiff base is weakened in the K intermediate. Taken together, it is likely that the interaction between the protonated Schiff base and the bound ion is broken upon photoisomerization.

Anion dependence was moderate in the L intermediate. The frequencies of the RR bands due to the vibrational modes in the polyene chain of retinal were similar for the chloride- and formate-bound forms, indicating that the polyene chain structure of the formate-bound form is similar to that of the chloride-bound form. The C=N stretching frequency was higher in the formate-bound form (1654 cm^−1^) than in the chloride-bound form (1651 cm^−1^), suggesting a difference in the hydrogen bond strength of the protonated Schiff base in the L intermediate. Crystallographic studies showed that the bromide ion moves across the Schiff base upon formation of the L intermediate for the bromide-bound form of NpHR,^47^ whereas the azide ion does not move in the azide-bound form.^7^ It is likely that the formate ion does not move across the Schiff base in the formate-bound form, similar to the azide ion in the azide-bound form. The observed difference in the hydrogen bond strength for the protonated Schiff base in the L intermediate is attributable to the difference in the position of the bound anion in the chloride- and formate-bound forms.

The rate of formation of the L intermediate is correlated with the hydrogen bond strength of the protonated Schiff base in the L intermediate, being faster in the state involving a stronger hydrogen bond. In the K-to-L transition of the chloride-bound form, the chloride ion moves to the cytoplasmic side across the retinal chromophore.^47,48^ The correlation between the L formation rate and the hydrogen bond strength of the protonated Schiff base suggests that the formation of the hydrogen bond during the transition lowers the energy barrier for L formation. We previously reported the anion dependence of the chromophore structure and dynamics in the halide-bound forms of NpHR.^13^ The present study revealed an even more remarkable anion effect on the structural changes in the chromophore pocket, elucidated by comparing RR data of the chloride-bound and formate-bound forms.

## V. CONCLUSION

We investigated the effect of the bound anion on protein structure and dynamics in the early stages of the NpHR photocycle. In the unphotolyzed state, the bound anion affects the π-conjugation of the retinal polyene chain and hydrogen bond strength. Due to the higher proton affinity of the formate ion compared to the chloride ion, the hydrogen bond of the protonated Schiff base in the formate-bound form is stronger than that in the chloride-bound form. This difference also affects the protein structure of the retinal pocket, as revealed by the intensity differences of the tryptophan UVRR bands. The UVRR intensities of the W16 and W18 bands decreased instantaneously upon photoexcitation of the formate-bound form whereas the intensity decreases were delayed with respect to the instrument response in the chloride-bound and anion-depleted forms. Comparison between the UVRR spectra of the wild type protein and the NpHR W127F and W229F mutants suggested that Trp127 and Trp222 are the most probable contributors to the observed spectral changes. The rate of structural rearrangement of the protein moiety following chromophore isomerization (20–30 ps) is very similar in the chloride-bound, formate-bound, and anion-depleted forms. Similar rates observed for other rhodopsins suggest that protein rearrangement within a few picoseconds following isomerization is common to the rhodopsins. The K intermediate showed weak anion dependence of the chromophore structure, indicating that the interaction between the protonated Schiff base and the bound ion is broken upon photoisomerization. This interaction is recovered in the L intermediate following translocation of the bound anion toward the protonated Schiff base. The present study elucidated the structural dynamics of the early stages of the NpHR photocycle and revealed how the interaction of the bound anion with the protonated Schiff base and the chromophore pocket affects protein structure and dynamics in the temporal range from picoseconds to microseconds.

## SUPPLEMENTARY MATERIAL

See the supplementary material for the titration of formate ion into anion-depleted NpHR, the procedure for obtaining transient RR spectra of anion-depleted NpHR, the effect of deuteration on the RR spectra of NpHR, comparison of UVRR intensities of chloride-bound, formate-bound, and anion-depleted NpHR, comparison of temporal intensity changes of the Raman bands between chloride-bound, formate-bound, and anion-depleted NpHR, and spectroscopic measurements of the NpHR mutants.
